# Surgical outcomes and predictors of complication in elderly patients with meningiomas

**DOI:** 10.1186/s41983-018-0005-3

**Published:** 2018-04-25

**Authors:** Ayman E. Galhom, A. A. Madawi, M. M. Ellabban

**Affiliations:** 1 Department of Neurosurgery, Faculty of Medicine, Port Said University, Port Said, Egypt; 2 Department of Neurosurgery, Faculty of Medicine, Suez Canal University Hospital, Ismailia, Egypt; 3 Department of Pathology, Faculty of Medicine, Port Said University, Port Said, Egypt

**Keywords:** Elderly meningioma, Surgery, Complication, Geriatric Scoring System

## Abstract

**Background:**

Surgical management of symptomatic meningioma in elderly is still a high-risk surgery due to increased incidence of complication rate. Many scoring systems have been proposed to expect the surgical risk and the outcome.

The study tries to assess cranial meningioma surgery in elderly using the Ibañez grade for complication rate and the Geriatric Scoring System (GSS) for the surgical outcome (GSS).

**Methods:**

A clinical and radiological data were studied retrospectively in 42 patients with a primary intracranial meningioma at or above the age of 65. Complication rate, surgical risk, and outcome were statistically analyzed.

**Results:**

The mean Geriatric Scoring System (GSS) score on admission was 15.4 ± 2.6. Ibañez grade of severe complication or death (grades III–IV) were experienced in 26.2% of patients. It was more common in male and in emergency cases, but it was significant in relation to the comorbidities (*P* < 0.004). Most patients had World Health Organization (WHO) grades I lesions, accounted for 85.7% of patients. MRI evidence of post-surgical residual was 14.4%, and 83.3% of patients had recurrence-free survival (RFS). The GSS score >16 were more frequent in the patient with RFS than those of < 16, and it was statistically significant (*P* < 0.06). Patient with Karnofsky performance status (KPS) < 70, the mean GSS was 14.5 and KPS > 70, the mean GSS was 18.9, and it was statistically significant (*P* < 0.002).

**Conclusion:**

The surgical technique for resection of elderly meningioma still had numbered cases of morbidity and mortality. The complication rate is related to preoperative co-morbidity and frequently associated with male and urgent surgery. Karnofsky score > 70, and RFS are favorable prognosis and related to GSS > 16 scores. The recurrence rate is usually attributed to high tumor grade and skull base tumor. Such scoring systems are valuable for elderly meningioma.

## Background

Intracranial meningioma originates from the arachnoid membrane cap cells where it covers the outer layer of the arachnoid mater and the arachnoid villi. In a primary intracranial tumor, it accounts for 30% (Wiemels and Wrensch 2010; Bartek et al. 2015). Woman is usually affected more than men (Kaul et al. 2015; Cohen-Inbar et al. 2011). Meningioma is a more frequent cranial tumor with peak incidence between the 6th and 7th decade (Schul et al. 2011). Due to advances in diagnostic brain imaging techniques and improvement in medical care, the humans’ lifespan and the incidence have been increased and are expected to rise more in the future (Chen et al. 2015; Bir et al. 2016). These incidences are underestimated due to the fact that a proportion of meningiomas are not surgically managed. Furthermore, autopsy and imaging studies have predicted subclinical meningioma up to 2.8% in women (Cohen-Inbar et al. 2011; Vernooij et al. 2007). Surgical treatment is not the solution for every case. Meningioma is usually asymptomatic, slowly growing small tumors without brain edema, especially in elderly patients. Such meningiomas mandate conservative clinical observation and radiologic follow-up. When meningioma becomes symptomatic, surgery is mandated (Kaul et al. 2015), especially for those with WHO grade I (Goldbrunner et al. 2016). In the older population, surgery was reported with overall complication rates ranged from 2.7 to 29.8% (Kaul et al. 2015). Reduced the functional status in older age has been mentioned as predisposing factors for poor outcome after surgery (Bartek et al. 2015; Poon et al. 2013). Furthermore, incomplete resection of meningioma usually needs multiple treatments, which increases morbidity and even mortality (Kaul et al. 2015; Poon et al. 2013). The growing incidence of this tumor in the elderly, the outcome, and the recurrence rate is not well documented (Schul et al. 2011; Bir et al. 2016; Caroli et al. 2005). The lack of subgroup analyses according to tumor location may yield confusing findings (Poon et al. 2013; Nakamura et al. 2005).

The aim of this retrospective study is to investigate the surgical risk and outcome of elderly meningioma. The study will employ a standardized two classification systems for the risk factor, operative complications, and surgical outcomes in elderly meningioma.

## Methods

### Patient population

We conducted a retrospective study on 42 patients who had a primary intracranial meningioma resection at Suez Canal University Hospital between 2006 and 2016 at or above the age of 65. The patient was reviewed by each patient’s case notes, operative records, out-patient appointments, and radiology reports. The extent of tumor resection was categorized according to the Simpson meningioma resection grade (Simpson 1957).

### Inclusion criteria

The study includes all patients aged 65 years and over admitted to and operated on by the Department of Neurosurgery with a minimum of 2 years follow-up.

### Exclusion criteria

Patients planned to be treated conservatively, recurrent meningioma, and biopsy were excluded.

### Preoperative

Ages, sex, clinical presentation, smoking habit, comorbidities, and medications (oral anticoagulant, an anti-epileptic drug, and long-term steroid) were analyzed. Patient age was defined as age at the time of operation. Imaging results were analyzed by experienced neuro-radiologists and include a definition of tumor location, size, a number of lesions, and presence of peri-tumoral edema. Initial MRI brain with contrast was done perioperatively (within 48 h of surgery). CT brain was done postoperatively to assess any bleeding incidence and degree of resection. During follow-up, MRI with contrast was done at 6, then 12 months after the operation. After that, it was annually performed for 5 years.

### Geriatric Scoring System

Geriatric Scoring System (GSS) score was assessed in all cases (Cohen-Inbar et al. 2011). In this scoring system, eight clinical and radiological parameters were included. These parameters include the following: tumor size, location, peri-tumoral edema, neurological deficits, Karnofsky performance status (KPS), associated diabetes, hypertension, and or lung disease. Each parameter consists of a score ranging from 1 to 3, and a total score range from 8 to 24.

### Indication for surgery

The indications for surgery included symptomatic tumors which are suitable for surgery and asymptomatic cases with evidence of tumor growth.

### Operative

Dexamethasone was administered a day before surgery for patients associated with brain tumor edema. All patients had preoperative prophylactic anticonvulsant phenytoin (5 mg/kg/day, oral) for 1 week. Patients will continue with anticonvulsant therapy when they presented with seizure preoperatively. Sodium valproate (15 mg/kg/day, oral) was initiated when phenytoin was contraindicated. Before surgery, 2 g ceftriaxone was administered. All patients received care in the intensive care unit postoperatively. Operative types (emergency or elective) were included.

Craniotomy for resection of meningioma was performed using standard micro-neurosurgical techniques for all patients. Tumor location included supratentorial (convexity, falcine/parasagittal), basal (sphenoid wing, olfactory/planum/sella), or infratentorial (posterior fossa, lateral tentorium). Histopathologic grading was classified according to WHO classification of meningioma grading (Goldbrunner et al. 2016; Louis et al. 2016), and Simpson grade of resection (Simpson 1957).

### Postoperative

Postoperative outcomes included the length of hospital stay, complications, mortality rate, and cause of death during follow-up. Functional outcome of improvement of neurological state, KPS were performed at 6-month, 1 year, and five years thereafter. Discharge destinations included “home”, or “rehabilitation center” according to the neurological recovery. New neurological deficits were defined as neurological signs demonstrable on clinical examination or new onset of seizures requiring lifelong anti-epileptic medications. Complications were classified according to the system proposed by Dindo et al. (2004) and further analyzed by Ibañez et al. (2011) and implemented by Poon et al. (2013) in his study.

The results of all postoperative neuroimaging (brain CT scans and MRI) were categorized as follows: complete resection, stable residual with no growth, a growth of the residual, evidence of tumor recurrence, and recurrence with new meningioma growth away from the original resection site.

### Statistical analysis

All statistical analyses were performed using SPSS (version 20). SPSS, Inc., Chicago, IL, USA). Frequency distributions and descriptive statistics were calculated for all variables. These include mean, median, and standard deviation. For univariate analysis, Pearson correlation, chi-square test, and Fisher exact test were used for comparison of non-parametric data. A *P* value of < 0.05 was considered significant.

## Results

### Patient characteristics

Forty-two patients with senile intracranial meningioma underwent intracranial meningioma resection in Suez Canal University Hospital center from 2006 to 2016 matched the inclusion criteria in this study.

Patient characteristics and demographic overview are listed in a Table 1. The “elderly” group included patients whose age lies between 65 and 78 years (mean 69.4 ± 4.3 years at a time of surgery). The majority of elderly 29 (69.1%) were women. The commonest presenting symptoms were a headache, convulsion, cognitive impairment, motor deficits, and visual disturbances.

**Table 1:** Demographic characteristics, preoperative status, and postoperative state

Demographic overview	No. (%)
Total patients	42 (100%)
Sex	
Female	29 (69.1%)
Male	13 (30.9%)
Clinical features	
Headache	25 (59.5%)
Vision impairment	4 (9.5%)
Seizure	13 (30.9%)
Gait disturbance	5 (11.9%)
Speech disorder	3 (7.1%)
Confusion	8 (19.1%)
Change in behavior	4 (9.5%)
Weakness/numbness	6 (14.3%)
Cerebellar symptoms	4 (9.5%)
Karnofsky Performance Status	
< 70 (26)	26 (61.9%)
≤ 70 (16)	16 (38.1%)
GSS	15.4 ± 2.6
Comorbidities	
Hypertension	30 (71.42%)
Diabetes	13 (30.95%)
Cerebrovascular disease	2 (4.8%)
Liver disease	4 (9.5%)
Heart disease	3 (7.1%)
Chronic lung disease	6 (14.3%)
Smoking	8 (19.1%)
Supratentorial	30 (71.4%)
Convexity	23 (54.8%)
Parasagittal	5 (11.9%)
Falx	2 (4.8%)
Skull base	10 (23.8%)
Olfactory groove	3 (7.1%)
Sphenoid wing/clinoidal	4 (9.5%)
Tuberculum sellae	2 (4.8%)
Tentorial	1 (2.4%)
Infratentorial	2 (4.8%)
Posterior fossa (CPA)	1 (2.4%)
Foramen magnum	1 (2.4%)
Size	
< 3 cm	5 (11.9%)
3–5 cm	23(54.8%)
> 5 cm	14 (33.3%)
Peritumoral edema	
Severe	20 (47.6%)
Mild	17 (40.5%)
Negligible	5 (11.9%)
Pathology WHO	
Grade I typical	36 (85.7%)
Transitional	17 (40. 5%)
Meningothelial	10 (23.8%)
Fibrous/fibroblastic	5 (11.9%)
Psammomatous	4 (9.5%)
Grade II a typical	5 (11.9%)
Grade III anaplastic	1 (2.4%)

*GSS* Geriatric Scoring System, *WHO* World Health Organization, *CPA* cerebellopontine angle tumor

**Fig. 1:**
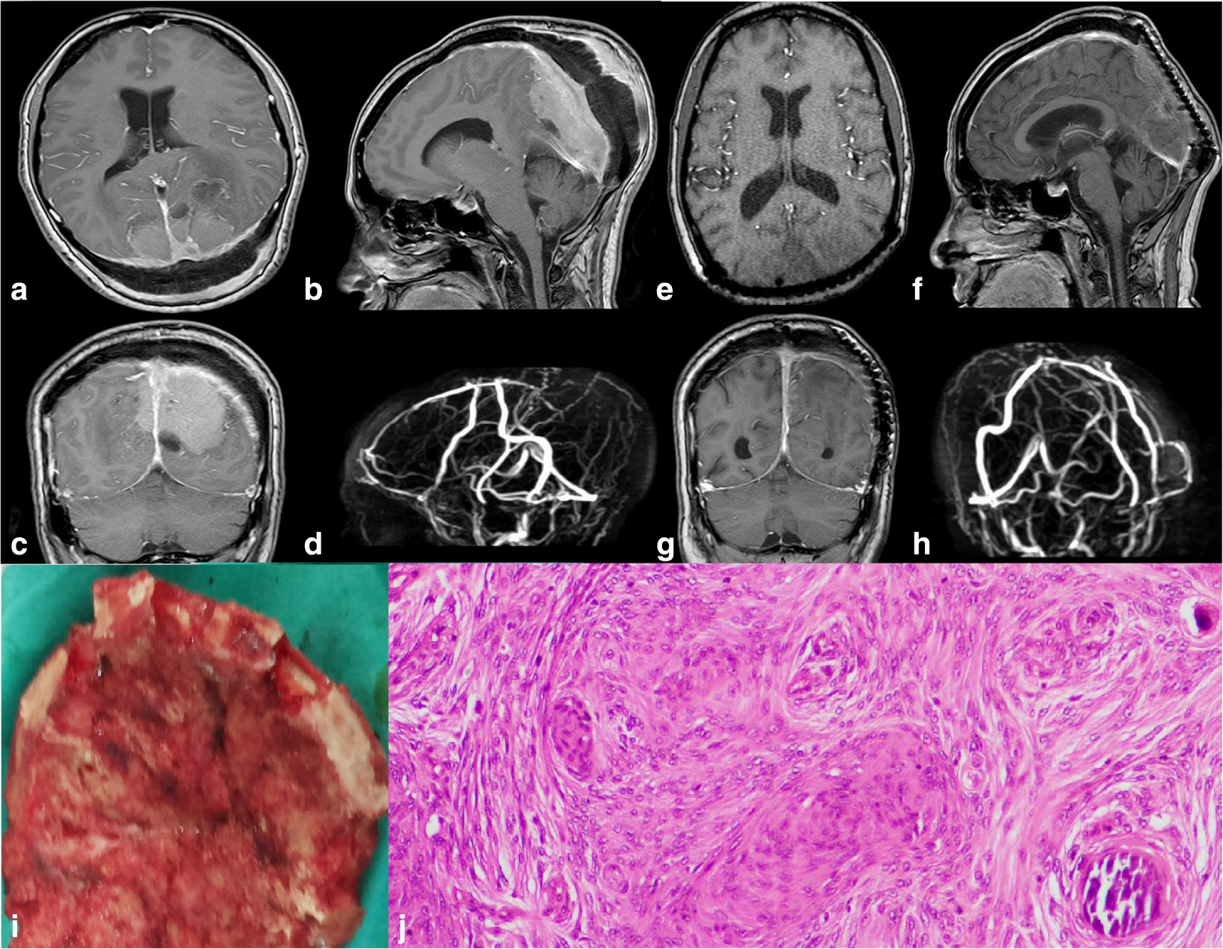
Meningioma in plaque situated in the parasagittal region. Preoperative image, MRI with contrast. **a** Axial view verified an enhanced occipital mass. **b** Sagittal view showed that tumor attached to the bone and involved the sagittal sinus. **c** Coronal view demonstrated mass lesion was crossing the midline bilateral in the parieto-occipital region more on the left side. The tumor was surrounded by mild edema. **d** It implicated the posterior aspect of the superior sagittal sinus and left transverse sinus in venography. Two year’s postoperative MRI with contrast. **e** Axial view revealed complete removal with nearly no mass lesion and decompressed occipital horn. **f** Sagittal view showed small residual mass parafalcine encasing superior sagittal sinus; the bony defect was closed by titanium mesh. **g** In coronal view, we discovered no mass lesion in the parieto-occipital region. **h** The posterior aspect of the superior sagittal sinus was opened. Geriatric Scoring System (GSS) score = 18. **i** It showed gross bony invasion of the tumor. **j** Slide represented syncytial meningioma with scatted psammoma bodies, WHO grade I

**Fig. 2:**
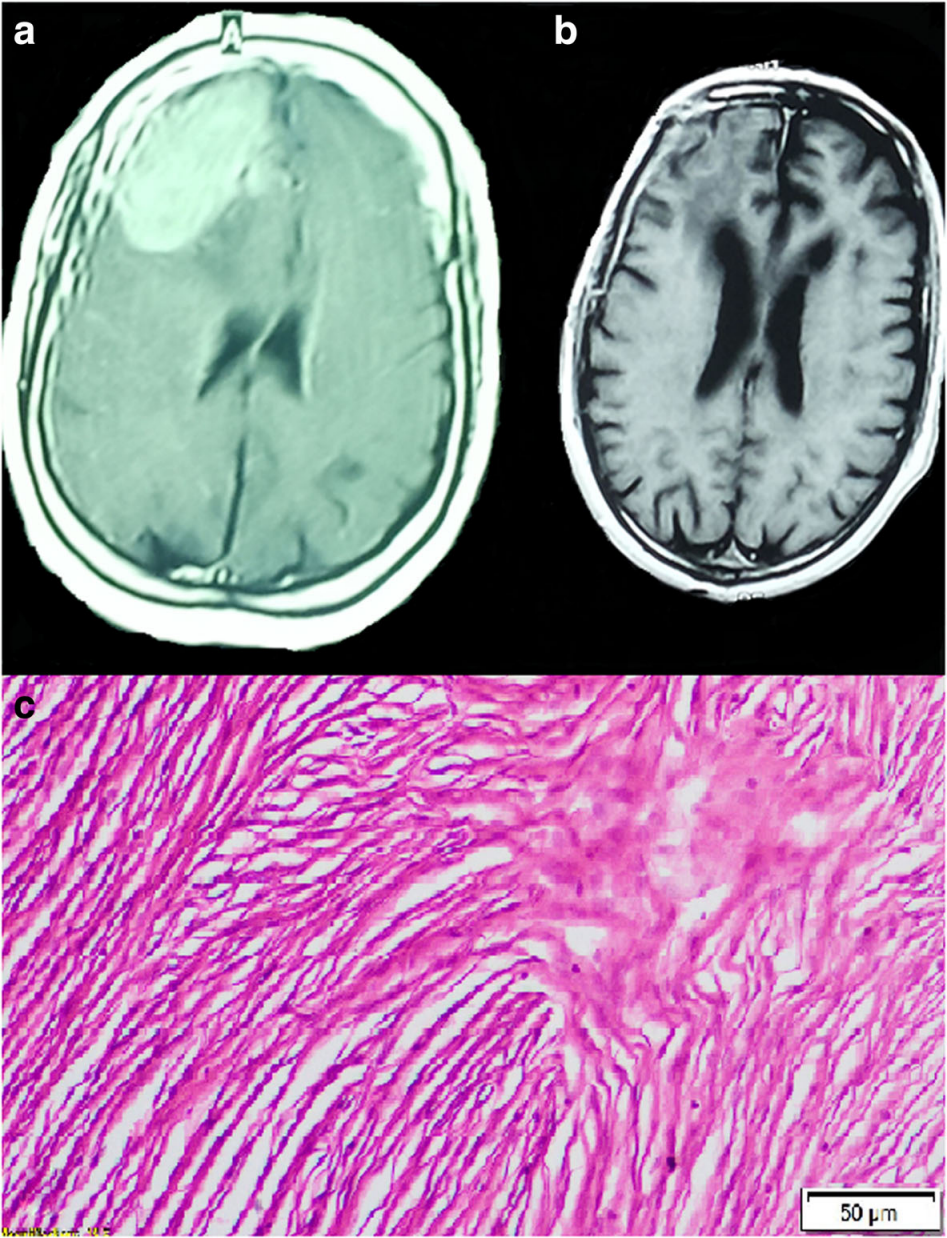
Convexity meningioma. **a** Preoperative image, MRI with contrast (axial) showed mass lesion in the right frontal region. The tumor was surrounded by mild edema. **b** One year postoperative MRI with contrast (axial) revealed complete removal of the mass. Geriatric Scoring System (GSS) score = 16. **c** The slide represented fibrotic meningioma, WHO grade I

**Fig. 3:**
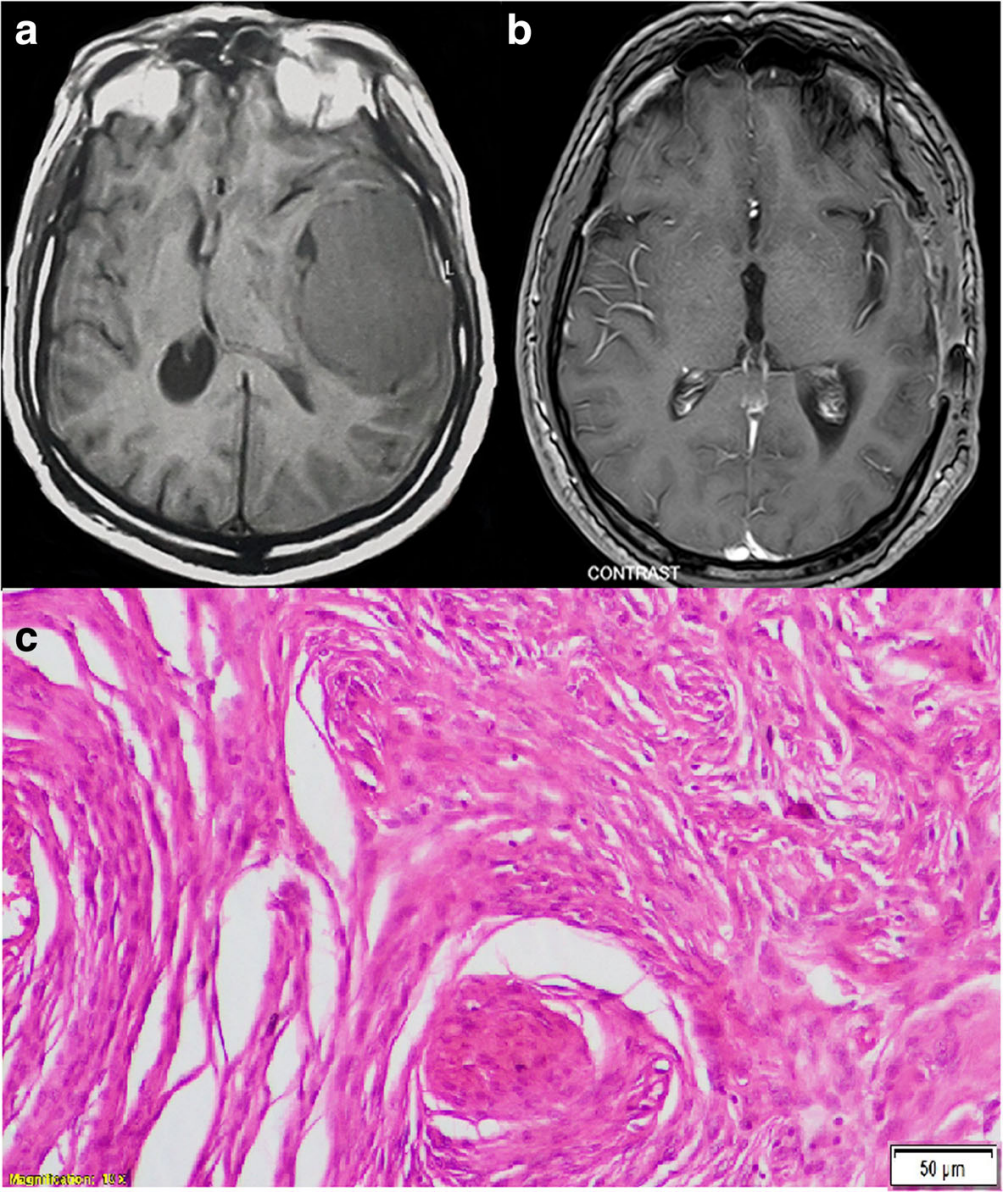
A convexity meningioma. **a** Preoperative image, MRI without contrast (axial) verified mass lesion in the right temporal region. The tumor was surrounded by mild edema. **b** Two year postoperative MRI with contrast (axial) demonstrated complete removal of the mass. Geriatric Scoring System (GSS) score = 16. **c** The slide represented transitional meningioma, WHO grade I

**Fig. 4:**
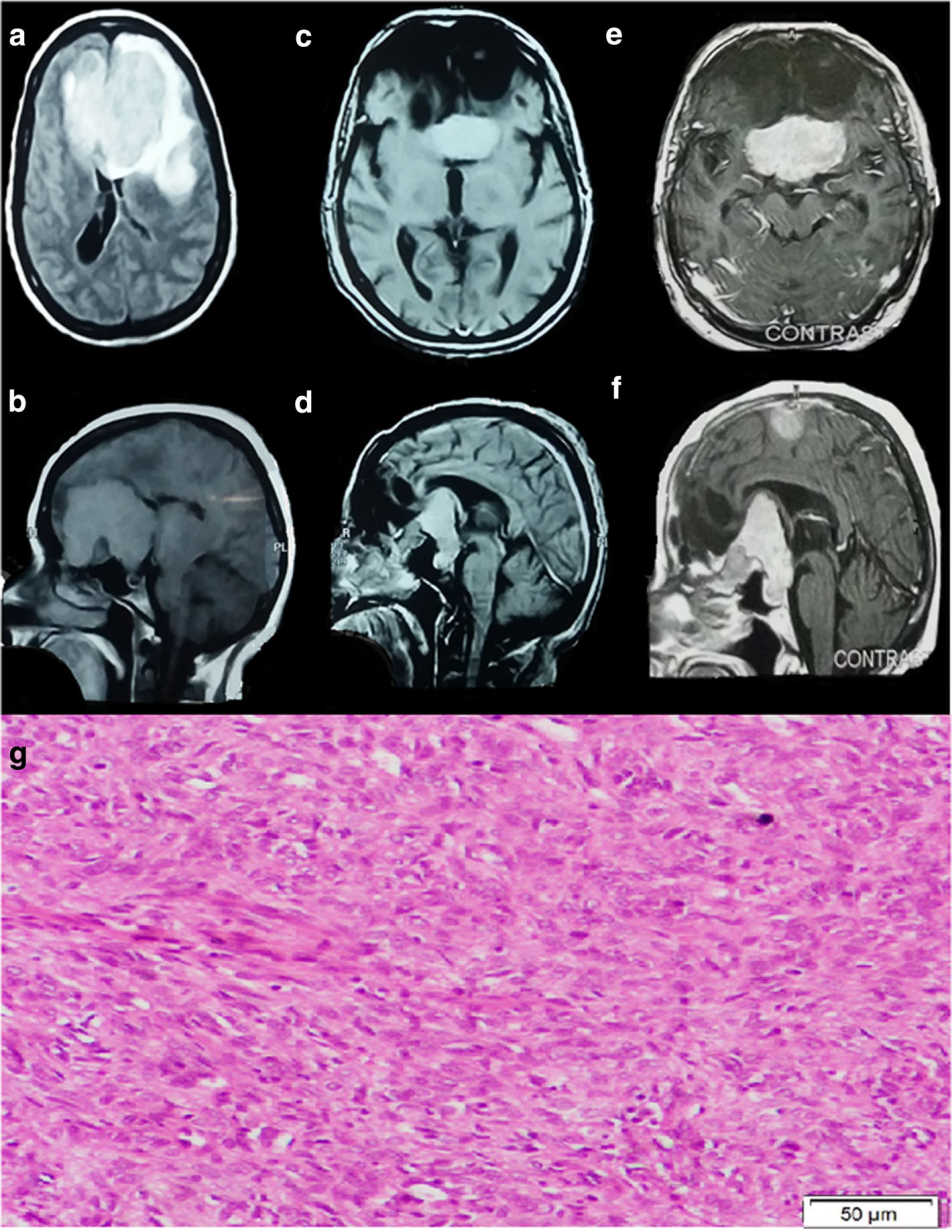
An olfactory groove meningioma. **a** Preoperative image, MRI with contrast (axial) showed mass lesion occupied the frontal region. **b** MRI with contrast (sagittal) demonstrated mass lesion arising from the floor of the anterior cranial fossa. The tumor was surrounded by marked edema. Geriatric Scoring System (GSS) score = 12 C). Four years postoperative MRI with contrast, **c** axial and **d** sagittal views showed recurrence of the tumor with extension into the third ventricle. The patient family refused surgery. Geriatric Scoring System (GSS) score = 11. **e** Six year postoperative MRI with contrast. **e** Axial cut represented progressive large tumor widening and splaying the arteries of the circle of Willis and the cerebral peduncles. **f** Sagittal view revealed the tumor was abutting the optic chiasma and reached the anterior and inferior aspect of the 3rd ventricle. It sent a distal metastasis into the left frontal cortical region. The family again refuse the surgery, and the patient were at high risk with repeated convulsion, and totally blind. **g** The slide represented an atypical meningioma WHO grade II. At power 10× the slide showed hypercellular tumor tissue formed of whorls of pleomorphic meningothelial cells showing pleomorphic and moderately hyperchromatic nuclei with frequent mitotic figures

**Fig. 5:**
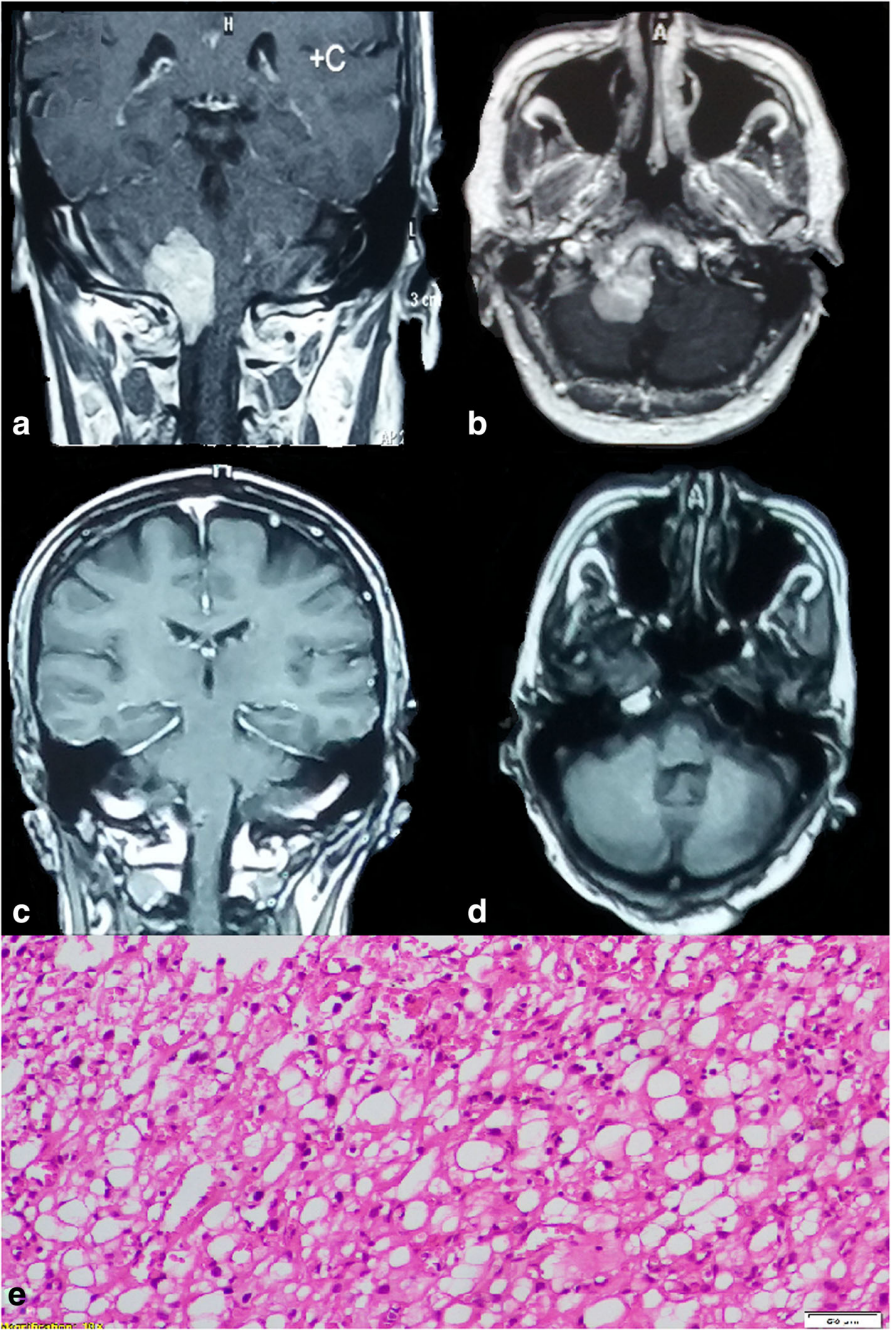
Foramen magnum meningioma. **a** Preoperative image, MRI with contrast (coronal) verified mass lesion compress the cervicomedullary junction and upper cervical cord. **b** MRI with contrast (axial) represented a ventral foramen magnum mass. **c** One year postoperative MRI with contrast (coronal) showed complete removal of the mass. **d** The same year MRI with contrast axial view demonstrated small ventral residual with complete medullary decompression. Geriatric Scoring System (GSS) score = 18. **e** The slide revealed a secretory variant of meningioma, WHO grade I

Radiological finding revealed 30 (71.4%) were classified as 5 (11.9%) parasagittal (Fig. 1) and 23 (54.8%) convexity (Figs. 2 and 3). In skull base 10 (23.8%), 4 (9.5%) sphenoid wing and 3 (7.1%) olfactory groove meningioma (Fig. 4). In infratentorial 2 (4.8%), foramen magnum meningioma constituted 2.4% of cases (Fig. 5).

The size of the lesion was as follows: 11.9% were less than 3 cm, 54.8% were 3–5 cm, and 33.3% were larger than 5 cm. A severe peri-tumoral edema on admission was noted in 47.6% of the cases, and 40.5% had mild edema. Elderly patients usually had a chronic illness; 71.4% had hypertension (HTN), 31% had diabetes mellitus (DM), and 14.3% of patients suffered from a respiratory disease as they were a smoker.

In clinical presentations found in our series included, 90.5% had some degree of neurological deficit. Functional status (KPS) was disabled to varying degree in 38.1%. The mean GCS score on admission was 15.4 ± 2.6, ranging 10–23, with a median of 15.0.

The majority of operations were performed as an elective surgery in 35(83.3%) patients, and the rest of cases were operated in the emergency due to rapid deterioration of the conscious level (Table 2).

**Table 2:** Operative and postoperative

Operative and postoperative	No. (%)
Operative type	
Elective	35 (83.3%)
Emergency	7 (16.7%)
Recurrence	7 (16.7%)
New growth	2 (4.8%)
On the same side of primary tumor	5 (11.9%)
Further treatment	8 (19.1%)
Re-surgery	2 (4.8%)
Radiation therapy	8 (19.1%)
Conventional radiation	3 (7.1%)
Radio-surgery	3 (7.1%)
Simpson grade	42(100%)
I	11 (26.2%)
II	25 (59.5%)
III	1 (2.4%)
IV	5 (11.9%)

After surgery, 16 (38.1%) patients experienced non-severe complication (grades I–II), and 9 (26.2%) patients (grades III–IV) had severe complication or death. Complication rates are presented in Table 3. Two patients had massive intraoperative brain edema and bleeding (patients 5 and 7). One patient (patient 5) died 5 days postoperative. Four patients (9.5%) had wound infection, seven patients (16.7%) developed new cranial nerve palsy, and one patient (2.4%) developed deep vein thrombosis. Complication was more common in male and in emergency cases but not statistically significant *P* < 0.12 and *P* < 0.23 respectively, but it was statistically significant in relation to the co-morbidities (*P* < 0.004).

**Table 3:** Clinical outcomes and complication after surgery

Outcome and complication	No. (%)
Mortality	6 (14.2%)
30 day	2 (4.8%)
1 year	1 (2.4%)
5 years	2 (4.8%)
Complications	
Grade I	14 (33.3%)
Grade II	2 (4.8%)
Grade IIIa	4 (9.5%)
Grade IIIb	2 (4.8%)
Grade IV	3 (7.1%)
Massive intraoperative bleeding and edema.	2 (4.8%)
Wound infection	4 (9.5%)
New hemiparesis, sensory changes	4 (9.5%)
New cranial nerve palsy	7 (16.7%)
Deep vein thrombosis.	1 (2.4%)
Discharge location	42 (100%)
Home ± rehabilitation	24 (57.1%)
Rehabilitation center	14 (33.3%)
Convalescence hospital	2 (4.8%)
Hospital persist	2 (4.8%)
Karnofsky score at 1 year	(*n* = 38)
> 70	23 (60.5%)
< 70	15 (39.5%)
Karnofsky score at 5 years	(*n* = 34)
> 70	20 (58.8%)
< 70	14 (41.2%)

The mean admission time at the hospital was 16.2 ± 10.3, and the mean admission at intensive care unit (ICU) was 7.4 ± 4.5 days. The average postoperative follow-up time was 5.7 ± 2.4 years with a range between 2.5 and 8.5 years. Imaging revealed that 30(71.4%) patients had no residual tumor findings in their observation imaging. Most patients had WHO grade I lesion, accounting for 36 (85.7%) patients (Figs. 1j, 2c, 3c, and 5e). The most common type is the transitional meningioma 17 (40.5%), followed by meningothelial 10 (23.8%), and fibrous/fibroblastic 5 (11.9%). An atypical grade II meningioma was found in 5 (11.9%) (Fig. 4g), and only 1 (2.4%) had an anaplastic type grade III meningioma.

For residual cases (14.4%), 4.8% of patients had small residual (Simpson grade IV), with no tumor growth on their follow up; 4.8% had conventional radiation; 2.4% had re-surgery; and 2.4% had gamma-knife radio-surgery (Figs. 1 and 5).

The recurrence rate was 16.7%. Two patients (4.8%) were found to have new growth of intracranial masses developed away from the primary incomplete resection site on their follow-up imaging. One patient needed re-surgery, and the other case was complicated by repeated convulsion but unstable for re-operation (Fig. 4). In those new growth cases, histopathological examination was atypical.

The recurrence rate at the site of resection was five (11.9%) patients. One case had Simpson grade II, one case had grade III, and three cases had grade IV resection (the last 4 cases were the rest of the residual cases). The histopathology for the recurrent cases were as follows: anaplastic, meningothelial meningioma, and atypical meningioma respectively. The only second case needed re-operation while the rest of cases did conventional radiation for recurrence.

For all residual cases, three cases had postoperative conventional radiotherapy, and three cases had gamma-knife radio-surgery radiation as they had financial resources’ for such expensive therapy. The tumor location for the residual cases was as follow: two cranial midline (one parasagittal, and one falcine) meningioma, and four skull base (two olfactory grooves, sphenoid wing, and tuberculum sellae) meningioma. The comparative data was not equal between those of gross total resection (grades I and II) (36 cases) and those of subtotal resection (grades III and IV) (6 cases) that we were not able to measure the difference. However, the recurrence rate was 3/36 for total resection cases comparative to 4/6 for the subtotal one.

Recurrence-free survival (RFS) was reported in 83.3% of cases. Mean RFS for the patients without (hypertension, diabetes mellitus, etc.) was 82 months, and with (hypertension, diabetes mellitus, etc.) comorbidities was 8 months, respectively, and it was statistically significant (*P* < 0.005). Mean RFS for patients without surgery were 86 comparative to 17 months for those with repeated surgery due to recurrence. GCS score > 16 were more frequent in the patient with RFS than those of < 16, and it was statistically significant (*P* < 0.06). Mean RFS for the patients with GSS score > 16 was 66 months, and the mean RFS for those with GSS score < 16 was 42 months. GSS score < 16 was more frequent in male and in high-grade meningioma, but it was not statistically significant (*P* < 0.12, *P* < 0.01). However, patients presenting with a higher GSS score had a significantly higher performance level and functioning 5 years after the surgery measured by KPS (Table 4). In patient with KPS < 70, the mean GSS was 14.5 and KPS > 70, the mean GSS was 18.9, and it was statistically significant *P* < 0.002.

**Table 4:** Univariant analysis of the complication, RFS, and GSS score versus different parameters

Outcome parameter examined	Different patient parameters	*P* value
Complication	Gender	0.12
	Clinical presentation	0.24
	comorbidities	0.004*
	KPS	0.023
	Operative type	0.23
	Operative time	0.14
	Peri-tumoral edema	0.03
	Pathology WHO	0.024
RFS	Gender	0.42
	Clinical presentation	0.45
	comorbidities	0.005*
	KPS	0.031
	Operative type	0.14
	Operative time	0.26
GSS	Gender	0.12
	Pathology WHO grade	0.01
	KPS	0.002*
	Operative type	0.15
	Operative time	0.14
	REF	0.06*

*KPS* Karnofsky Performance Status, *WHO* World Health Organization, *RFS* Recurrence-free survival, *GSS* Geriatric Scoring System

*P value was clinically significant

During the follow-up period, there were five deaths. One patient had massive edema and hemorrhage and died 5 days postoperative, one patient, a 75-year-old man, developed conscious level deterioration and convulsion 5 years after surgery. He had residual and he was not fit for surgery and died due to pneumonia unresponsive in the intensive care units. The other patient died postoperatively due to severe venous infarction in a large parasagittal meningioma 15 days postoperation. The last two cases died to an unrelated condition of myocardial infarction and cerebrovascular accident.

## Discussion

Intracranial meningiomas are the commonest benign tumor in elderly patients (Wiemels and Wrensch 2010). Many studies had been discussed the risk factors affecting the surgical outcome in such age group (Bartek et al. 2015; Kaul et al. 2015; Chen et al. 2015; Poon et al. 2013; Caroli et al. 2005). Patients aging 70 years or more, KPS less than 70, duration of surgery more than 4 h, and extent of tumor resection (Chen et al. 2015; Poon et al. 2013) are suggested as predictive factors for surgical outcome (Wiemels and Wrensch 2010; Schul et al. 2011; Bir et al. 2016). In this study, we performed surgery for the patient more than 65 years old. Nakamura et al. found that there is no difference in complication rate between two age groups patients with 65 or 70 years respectively (Nakamura et al. 2005). On the other hand, surgical outcome and degree of tumor resection is another issue discussed in such age group (Bir et al. 2016). Many patients are asymptomatic, as the tumor figure 21% of all primary intracranial tumors, to be risen above 40% in the autopsy (Cohen-Inbar et al. 2011). In elderly, tumor behavior, hormonal profile, vascularity, histopathological type, and grade are different from young patients (Poon et al. 2013; Hussain and Erdek 2014; Roser et al. 2007).

In younger patients, surgery even in a non-symptomatic patient is considered curative especially if increase in size is monitored during follow-up. In elderly, however, conservative management is recommended even in large tumor unless it causes symptoms that mandate intervention (Bartek et al. 2015).

Asymptomatic meningiomas are usually followed up without surgery, especially in elderly where there is a higher incidence of both morbidity and mortality (Nayeri et al. 2016). From the above statement; many scoring systems have been proposed to expect the surgical risk and the outcome. SKALE [sex, Karnofsky, American Society of Anesthesiology, location, edema] (Sacko et al. 2007), Clinical and Radiological Grading System (Caroli et al. 2005), and GSS (Cohen-Inbar et al. 2011) have been proposed by many studies. However, GSS gains some popularity due to its simplicity and depends on its analysis system on the patient, the tumor with their relation to the outcome (Cohen-Inbar et al. 2011; Bir et al. 2016). Studies revealed that patient with score ≥ 16 has a favorable prognosis (Cohen-Inbar et al. 2011; Bir et al. 2016). Bir et al. (Bir et al. 2016) suggest a significant relationship between GSS score, tumor recurrence, and usage of antiepileptic medication after surgery. Incomplete resection is associated with recurrence (Wiemels and Wrensch 2010; Schul et al. 2011; Bir et al. 2016; Poon et al. 2013). In this study, complete resection of meningioma (Simpson grades I and II) was achieved in 85.7% comparable to 84% in one study, and subtotal resection (grades III and IV) reached 14.3% comparable to 16% of the same study (Bir et al. 2016).

Karnofsky score and RFS were statistically significant in relation to high GSS score. However, male and high meningioma grade were associated with low GSS score but not statistically significant. Some studies (Cohen-Inbar et al. 2011; Bir et al. 2016) found such relationship. In a long cohort study in patients with intracranial meningioma, elderly patients have a poorer outcome compared to the younger one (Patil et al. 2010).

Surgical technique for resection of elderly meningioma was not a low-risk surgery. Literature revealed a wide range of mortality that extended from 1.2 up to 45% in some study (Bartek et al. 2015; Chen et al. 2015; Poon et al. 2013; Sacko et al. 2007; Patil et al. 2010). In this study, it reached 11.9%. Concerning morbidity, development of new onset neurological deficit reported up to 29.8% in some studies (Bateman et al. 2005) and 21.4% in this study. Attempts for total resection in skull base meningiomas are associated with higher incidence of morbidity (Bir et al. 2016; Van Alkemade et al. 2012). Poon MT et al. (2013) did not find much difference between the group of total resection from those with subtotal removal. Roser et al. (2007) and Poon et al. (2013) found that complication is more frequent in skull base tumor. Furthermore, Poon et al. (2013) demonstrated that convexity, parasagittal and falcine groups were less likely to develop grade III and IV complications. Many long cohort studies have compared the rate of complication between elderly and non-elderly patients with meningioma after surgery. All studies found nearly that elderly patients had a double complication rate comparable to a non-elderly one. Patil et al. (2010) study in 1281 patients found that incidence of complication was 29.8% for elderly versus 13.1% for non-elderly one. Bateman et al. (2005) in a study from 1998 to 2002, they analyzed the surgical outcome of 8861 patients with meningioma. They divided the patients into 2304 elderly patients (70 years or older) comparable to 6557 patients younger (from 20 to less than 70 years). The data revealed nearly double rate parameter in each category as follow: the in-hospital mortality rate (4.0% versus 1.1%, *P* < 0.001), the rehabilitation facility discharge rate (53.2% versus 16.6%, P < 0.001), and the longer mean length of stay (7.2 versus 5.1 days; *P* < 0.001) in elderly and non-elderly respectively. Connolly et al. (2015) study between 2010 and 2012, and during their analysis of 2216 patients. They found elderly patient (≥ 70 years) developed 1 or more perioperative complication in 55% comparable to 39% of non-elderly patients. Grossman et al. (2011) studied 5717 patients older than 65 years old and found that the overall complication was 39.4% and mortality rate 3.2% double the non-elderly number of Bateman et al. (2005)

Gender was not associated with complication after surgery; such statement was found in a large retrospective study of Grossman et al. (2011); in this study, the majority of the patients were female (66.6%). However, Poon et al. (2013) and Connolly et al. (2015) found that complication rate was more frequent in female. Connolly et al. (2015) attributed such female tendency due to large female population in their study (70%). However, Patil et al. (2010) reported in their long cohort study of elderly and non-elderly patients with meningioma that it was more frequent in males with 98% for elderly comparative to 94% for non-elderly patients. In this study, the complication was more common in male and emergency cases but was not statistically significant. Grossman et al. (2011) and Poon et al. (2013) found emergency procedure was the predictor of severe complication due to respiratory trouble postoperative which affected the outcome. For monitoring complication, we used Ibañez classification grade (Ibañez et al. 2011) for complication which was adapted from other medical field and was recommended by Bartek et al. (2015). One of the most common complications after surgery is seizures. Meningioma causes seizure in various ways including the following: focal cortical hypoxia, edema and mass effect, or cortical damage after surgery (Wyllie et al. 1998). Perioperative antiepileptic drugs prevent operative and postoperative seizures and seizure-induced brain damage. Prophylactic Dilantin and sodium valproate are usually used with no difference between two drugs (Zhang et al. 2015). However, recent meta-analyses did not support such measurement of prophylaxis (Patil et al. 2010; Connolly et al. 2015). In this study, the incidence of seizure was (30.9%) where it was reported 11.8% elsewhere (Connolly et al. 2015). Conventional radiotherapy or radiosurgery is potential option for treatment of meningioma in elderly patients who present without symptoms or unstable for surgery. Many studies have been discussed tumor control with the least complication for radiosurgery in elderly patient (Bartek et al. 2015; Goldbrunner et al. 2016; Kreil et al. 2005). In the study, 7.1% of patient had adequate response to radiosurgery.

In 1957, Simpson classification for meningioma recurrence had been proposed that the extent of resection is the main cause of recurrence of meningioma (Simpson 1957). WHO grade of meningiomas could affect the recurrence rate; grade I has a low recurrence rate of 7% within 5 years of resection (Nayeri et al. 2016).In elderly, the recurrence may be higher due to several factors including the following: associated comorbidities (Bartek et al. 2015), location of the tumor (skull base), large tumor (Bir et al. 2016; Patil et al. 2010), and high-grade meningioma (Goldbrunner et al. 2016; Louis et al. 2016). Repeated cranial resection of meningioma is associated with low recurrence rate (Bir et al. 2016; Black et al. 1998). The recurrence rate was reported to be 7–16% (Bir et al. 2016; Nayeri et al. 2016).The study showed recurrence rate (16.7%). This number may be attributed to higher number of skull base tumor, high-grade tumor, and the relation of the tumor to the neurovascular bundle. In one study, the tumor recurrence according to grade (grades I–IV, 5, 9, 45, and 50%, respectively) (Bir et al. 2016).

### Limitation

The study had some limitation including a small number of cases, its retrospective nature, and short-term outcome in some patients. Many prognostic factors have been suggested for tumor recurrence and hence the patient outcome. These include the higher mitotic index, cell proliferation (Ki-67) index, and existing neoplastic dura cells around the craniotomy site (Wiemels and Wrensch 2010; Bir et al. 2016; Simpson 1957).

## Conclusions

Adequate preoperative preparations and using GSS as outcome predictor are the mainstay of patient selection for surgery in elderly meningioma. Ibañez scoring system for complication classification is another important scoring categorization for predict outcome from complication. Further study is recommended for surgical decision making is such high-risk age group.
